# Genomic alterations and associated outcomes in patients with PSMA-positive metastatic castration-resistant prostate cancer treated with ^177^Lu-PSMA-617

**DOI:** 10.1093/oncolo/oyaf358

**Published:** 2025-10-27

**Authors:** Justine Panian, Nicholas C Henderson, Daniel Herchenhorn, Pedro C Barata, Mehmet Asim Bilen, Laura Graham, Elisabeth Heath, Clara Hwang, Avery Supernois, Deepak Kilari, Bicky Thapa, Vadim S Koshkin, Tanya Jindal, Jones T Nauseef, Alexandra Sokolova, Taylor Amery, Yousef Zakharia, Michael T Schweizer, Ruben Raychaudhuri, Zachery R Reichert, Tanya Dorff, Andrew J Armstrong, John Wang, Ajjai Alva, Rana R McKay

**Affiliations:** University of California San Diego, Department of Medicine, San Diego, CA 92037, United States; University of Michigan, Department of Medicine, Ann Arbor, MI 48109, United States; University of California San Diego, Department of Medicine, San Diego, CA 92037, United States; University Hospitals Seidman Cancer Center, Department of Medicine, Cleveland, OH 44106, United States; Winship Cancer Institute of Emory University, Department of Medicine, Atlanta, GA 30322, United States; University of Colorado Anschutz Medical Campus, Department of Medicine, Aurora, CO 80045, United States; Wayne State University, Department of Medicine, Detroit, MI 48201, United States; Henry Ford Health System, Department of Medicine, Detroit, MI 49201, United States; Henry Ford Health System, Department of Medicine, Detroit, MI 49201, United States; Medical College of Wisconsin, Department of Medicine, Milwaukee, WI 53226, United States; Dana-Farber Cancer Institute, Department of Medicine, Boston, MA 02215, United States; University of California San Francisco, Department of Medicine, San Francisco, CA 94143, United States; University Hospitals Seidman Cancer Center, Department of Medicine, Cleveland, OH 44106, United States; University of California San Francisco, Department of Medicine, San Francisco, CA 94143, United States; Weill Cornell Medicine, Department of Medicine, New York, NY 10065, United States; Oregon Health & Science University, Department of Medicine, Portland, OR 97239, United States; Oregon Health & Science University, Department of Medicine, Portland, OR 97239, United States; University of Iowa Holden Comprehensive Cancer Center, Department of Medicine, Iowa City IA, 52242, United States; University of Washington/Fred Hutchinson Cancer Center, Department of Medicine, Seattle, WA 98109, United States; University of Washington/Fred Hutchinson Cancer Center, Department of Medicine, Seattle, WA 98109, United States; University of Michigan, Department of Medicine, Ann Arbor, MI 48109, United States; City of Hope Comprehensive Cancer Center, Department of Medicine, Duarte, CA 91010, United States; Duke Cancer Institute Center for Prostate and Urologic Cancer, Duke University, Department of Medicine, Durham, NC 27710, United States; Duke Cancer Institute Center for Prostate and Urologic Cancer, Duke University, Department of Medicine, Durham, NC 27710, United States; University of Michigan, Department of Medicine, Ann Arbor, MI 48109, United States; University of California San Diego, Department of Medicine, San Diego, CA 92037, United States

**Keywords:** genomics, metastatic, prostate, ^177^Lu-PSMA-617

## Abstract

**Background:**

^177^Lu-PSMA-617 is approved for patients with metastatic castration-resistant prostate cancer (mCRPC). Although treatment is associated with improved outcomes, not all patients benefit and response is heterogeneous. We aim to characterize genomic alterations associated with benefit to ^177^Lu-PSMA-617.

**Materials and Methods:**

This study used the Prostate Cancer Precision Medicine Multi-Institutional Collaborative Effort (PROMISE) clinical-genomic database (*n* = 2445). The primary endpoint was ≥50% PSA decline (PSA_50_) from baseline with ^177^Lu-PSMA-617 in molecular subgroups. Secondary endpoints included 90% PSA decline (PSA_90_). Associations were assessed using Fisher’s exact test and Cox regression in multivariable analysis.

**Results:**

We identified 183 mCRPC patients treated with ^177^Lu-PSMA-617. Median number of prior lines of mCRPC therapy was 3. Overall, PSA_50_ was 49%, median progression-free survival was 7.6 months, and median overall survival was 13.9 months. *NF1* (*n* = 8) and *FOXA1* alterations (*n* = 5) were associated with increased PSA_50_ (88% vs 47%, *P* = .03 for *NF1*; 100% vs 47%, *P* = .03 for *FOXA1*). Among CRPC sequenced tumors (*n* = 119), androgen receptor (AR) alterations (*n* = 58) were associated with lower PSA_50_ (38% vs 60%, *P* = .03). While any tumor suppressor genes (TSG) (*PTEN*, *TP53*, *RB1*) (*n* = 109) or *TP53* (*n* = 83) alteration were associated with lower PSA_90_ (*P* = .02 for both), *NF1* (*n* = 8), and *FOXA1* alterations were associated with higher PSA_90_ (*P* = .03 and *P* = .003, respectively).

**Conclusions:**

This analysis identifies potential genomic predictors of response to ^177^Lu-PSMA-617, with *NF1* and *FOXA1* alterations associated with favorable outcomes and *AR* and TSG alterations with diminished response. These hypothesis-generating findings suggest genomic profiling may inform selection for PSMA-targeted therapy and warrant prospective validation in larger cohorts.

Implications of Practice In this study, we evaluate the use of genetic markers to predict response to treatment with ^177^Lu-PSMA-617 in patients with metastatic prostate cancer. We identified that alterations in the AR and TSG were associated with a worse response to ^177^Lu-PSMA-617, which *FOXA1* and *NF1* alterations were associated with improved outcomes. These data are hypothesis generating and warrant validation in larger studies. Identifying predictive markers to ^177^Lu-PSMA-617 can better optimize treatment selection for this therapy.

## Introduction

^177^Lu-PSMA-617 is a β-emitting radioligand therapy approved for patients with metastatic castrate-resistant prostate cancer (mCRPC) who were previously treated with taxane chemotherapy and androgen receptor pathway inhibitors (ARPI).^1^ This agent has a small molecule ligand (PSMA-617) that binds with high affinity to prostate-specific membrane antigen (PSMA), conjugated to the radionuclide lutetium-177 (^177^Lu). The efficacy of ^177^Lu-PSMA-617 was demonstrated in the VISION trial, which included patients having received prior chemotherapy and an ARPI showing significant improvements in overall survival (OS), progression-free survival (PFS), objective response rate (ORR), and prostate-specific antigen (PSA) response rate compared to standard of care.^2^ Furthermore, the PSMAFore trial demonstrated the benefit of ^177^Lu-PSMA-617 in the pre-chemotherapy setting with significant improvements in PFS, ORR, and PSA response, though no benefit in OS was observed.

Although treatment with ^177^Lu-PSMA-617 was associated with improved PFS in the overall population in both the VISION and PSMAFore trials, not all patients derive the same benefit, with primary and acquired resistance remaining a significant challenge. While eligibility criteria for ^177^Lu-PSMA-617 therapy rely primarily on the presence of PSMA PET expression, these imaging criteria alone do not fully predict treatment response. Currently, no prospectively validated molecular biomarkers exist to guide optimal patient selection for ^177^Lu-PSMA-617 therapy. In the VISION trial, notable objective (51%) and PSA responses (46%) were observed, yet a subset of patients (13%) had progressive disease as their best response, and the majority eventually developed disease progression. Though the trial resulted in statistically significant improvements in OS (median 15.3 vs 11.3 months, hazard ratio [HR] 0.62) and radiographic PFS (median 8.7 vs 3.4 months, HR 0.40), these benefits were modest and durable responses limited. These outcomes underscore the urgent need for better predictive biomarkers to optimize therapy selection and maximize clinical benefit.

Analysis of early PSA dynamics further highlights the variability in outcomes to ^177^Lu-PSMA-617.^3^ Specifically, patients who had a PSA increase within the first 12 weeks (29%) showed limited benefit (median radiographic PFS 5.8 months and OS 9.8 months), while those with PSA declines of 50%-90% (28%) had improved outcomes (median radiographic PFS 11.3 months and OS 18.3 months). The most favorable prognosis was observed in patients with early extreme PSA decline (PSA decline >90%, 15%), achieving median radiographic PFS of 20.3 months with median OS not yet reached. These dramatic differences in survival outcomes based solely on early PSA response patterns underscore the profound heterogeneity in treatment benefit and the critical importance of identifying molecular determinants of response and resistance.

Limited data have analyzed associations between ^177^Lu-PSMA-617 efficacy and clinical, laboratory, and imaging parameters.^4–15^^,^^16–18^ A predictive nomogram developed from VISION trial data incorporated multiple variables including SUVmax, time since diagnosis, laboratory values (lactate dehydrogenase, alkaline phosphatase, hemoglobin, lymphocyte count), and presence of PSMA-positive lymph nodes or liver metastases.^19^ While this model demonstrated good predictive accuracy (C-index 0.68 for radiographic PFS), it notably lacked molecular information.

Recent analysis from the PSMAFore trial has begun to address this gap, revealing that higher baseline ctDNA fraction correlates with shorter radiographic PFS regardless of treatment, while early ctDNA clearance predicts improved outcomes. Specific genomic alterations, including 8q amplifications, *AR* amplifications, and *TP53* deleterious mutations, were identified as prognostic biomarkers associated with poorer responses to ^177^Lu-PSMA-617.

Given these findings, we embarked on this real-world analysis to investigate molecular alterations associated with benefit to ^177^Lu-PSMA-617. We hypothesized that patients with select gene alterations, particularly in tumor suppressor genes (TSG) and androgen receptor (AR) pathways, would experience worse outcomes following ^177^Lu-PSMA-617 treatment. Our project aimed to characterize molecular determinants of response and resistance, addressing a critical unmet need for optimized patient selection. These preliminary data are hypothesis-generating and may potentially inform future prospective validation studies.

## Design and methods

### Study design and patient selection

We conducted a retrospective analysis utilizing the Prostate Cancer Precision Medicine Multi-Institutional Collaborative Effort (PROMISE) clinical-genomic database, which includes deidentified clinical and genomic data from patients with advanced prostate cancer (metastatic hormone sensitive or castration resistant) (*n* = 2445) involving 11 academic US sites. Patients had germline and somatic genomic testing (tissue, blood, and/or germline) through CLIA-certified commercially available platforms during routine clinical care. For the PROMISE registry, clinical and genomics data were extracted from electronic medical records using a standardized RedCap database. This study was approved by local institutional review boards at participating sites per institutional policy and the Declaration of Helsinki.

We included mCRPC patients with at least one somatic sequencing test and at least one dose of ^177^Lu-PSMA-617 (*n* = 183). Both tumor tissue and ctDNA sequencing assays were utilized; however, ctDNA tests were only included if at least one pathogenic or likely pathogenic somatic alteration was detected. Patients with only available germline testing were excluded.

Quality control was performed by a physician with genomic expertise on at least 10% of entries from each institution. Any entries requiring clarification were flagged and sent back to the original site for resolution. Following this 2-stage verification process, a genomics expert at the central site (University of Michigan) reviewed the data to confirm that only pathogenic or likely pathogenic alterations were included in the analysis.

## Outcome measures

Data regarding patient demographic characteristics, PSA values, treatment types, genomic profile, and assay type were extracted from the database. All baseline, on-treatment, and progression PSA values that were available through clinical standard of care testing were collected throughout the duration of ^177^Lu-PSMA-617 therapy. In patients with more than one genomic testing, tissue testing was prioritized over ctDNA testing and testing performed on tumor sample procured more proximal to the timing of ^177^Lu-PSMA-617 initiation was prioritized.

The primary objective was to compare the rates of PSA_50_ decline from baseline with ^177^Lu-PSMA-617 between patients with and without select molecular alterations. PSA response rate was defined as the proportion of patients with a 50% or greater decline in PSA from baseline (PSA_50_), without a requirement for confirmatory testing. As a secondary endpoint, we also examined PSA_90_, defined as the proportion of patients achieving a 90% or greater decline in PSA from baseline. Additional secondary endpoints included clinical or radiographic PFS and OS. PFS was defined as the time from the first dose of ^177^Lu-PSMA-617 to the time of clinical or radiographic progression using Prostate Cancer Working Group 3 principles. OS was defined as the time from the first ^177^Lu-PSMA-617 to death or last follow-up. For genomic data, the mutation groups included TSG alterations (ie, *PTEN*, *TP53*, *RB1*), *AR* alterations (AR mutations and amplifications), and homologous recombination repair (HRR) alterations (*BRCA1, BRCA2, ATM, ATR, BRIP1, BARD1, CDK12, CHEK1, CHEK2, FANCA, FANCL, PALB2, PPP2R2A, RAD51B, RAD51C, RAD51D, NBN, MLH1, MRE11A*, *RAD54L*).^20^^,^^21^ Microsatellite instability (MSI) was defined as “unstable” by molecular assessment, which included high instability detected through PCR-based or next-generation sequencing methods demonstrating alterations in microsatellite regions.

For statistical analysis, PSA_50_ and PSA_90_ were calculated among patients with at least one pre-treatment baseline value (collected within 90 days of treatment initiation) and one post-treatment initiation PSA value. Of the total cohort (*n* = 183), 164 patients were evaluable for the primary endpoint based on having both pre- and post-treatment PSA measurements. PSA_50_ and PSA_90_ rates were compared using Fisher’s exact test, and confidence intervals (CI) were found using the Wilson method. Overall, 178 and 168 patients with sufficient follow-up data were evaluable for OS and PFS, respectively. OS and PFS curves were constructed using the Kaplan-Meier method, with comparisons between gene-altered and non-altered groups performed using log-rank tests. Univariate OS and PFS HR between groups were calculated using Cox proportional hazards models. Adjusted HRs and 95% CIs for OS and PFS, comparing gene-altered vs non-altered groups, were derived using Cox proportional hazards models with adjustment for relevant patient and disease characteristics at ^177^Lu-PSMA-617 initiation.

We determined there to be a significant association between an alteration and PSA_50_ response if the *P*-value from a Fisher’s exact test was less than .05. Corrections for multiplicity were not performed due to the limited available sample size and low power to detect any associations. Given the observed frequencies of genetic alterations among our cohort of 183 patients and assuming that 3 of these alterations (TSG or *AR*) were associated with differences in PSA_50_ response rates (2 with a PSA_50_ response odds ratio [OR] of 0.75 and 1 with a PSA_50_ response OR of 0.33), we calculated that our approach had 81% power to detect a PSA_50_ response difference in at least one of the alterations among the alterations considered. Given the available sample size, we had much more limited power to detect more than one alteration, with 47% power to detect 2 or more alterations associated with PSA_50_ response and 19% power to detect 3 such alterations.

## Results

### Baseline demographics

We identified 183 patients with PSMA PET positive mCRPC treated with at least one dose of ^177^Lu-PSMA-617 (Figure S1). The median age was 72 years, 45 (25%) patients were non-white, and 73 (40%) had de novo metastatic disease at diagnosis. At ^177^Lu-PSMA-617 initiation, the median PSA was 18.4 ng/mL. Metastatic disease sites included lymph nodes (64%, *n* = 117), bone (87%, *n* = 160), lung (13%, *n* = 24), and liver (14%, *n* = 26) (Table 1).

**Table 1.** Baseline characteristics of patients receiving ^177^Lu-PSMA-617 therapy.

Characteristic	*N* = 183
**Median age (years)**	72
**Race, No (%)**	
** White**	138 (75%)
** Asian**	4 (2%)
** Black**	28 (15%)
** Mixed race**	2 (1%)
** Other**	11 (6%)
**Ethnicity, No (%)**	
** Hispanic**	9 (5%)
** Non-Hispanic or unknown**	174 (95%)
**Gleason grade, No (%)**	
** <8**	59 (32%)
** 8-10**	95 (52%)
** Missing**	29 (16%)
**Median PSA at diagnosis (ng/mL)**	18.4
**De novo metastatic disease, No (%)**	
** Yes**	73 (40%)
** No**	110 (60%)
**Local therapy prior of ^177^Lu-PSMA-617, No (%)**	
** Prostatectomy**	85 (46%)
** Local radiation**	97 (53%)
**Sites of metastatic disease at time of ^177^Lu-PSMA-617, No (%)**	
** Lymph Node**	117 (64%)
** Bone**	160 (87%)
** Lung**	24 (13%)
** Liver**	26 (14%)
**Chemotherapy Naïve, No (%)**	
** Yes**	28 (15%)
** No**	155 (85%)
**Prior lines of therapy in mCRPC, No (%)**	
** 0**	11 (6%)
** 1**	24 (13%)
≥ **2**	148 (81%)
**Prior lines of ARPI, No (%)**	
** 0**	3 (2%)
** 1**	76 (41%)
≥ **2**	104 (57%)
**Prior lines of Taxane, No (%)**	
** 0**	28 (15%)
** 1**	69 (38%)
≥2	86 (47%)
**> 1 ARPI and > 1 Taxane, No (%)**	
** Yes**	52 (28%)
** No**	131 (72%)
**Treatments received concurrently with of ^177^Lu-PSMA-617, No (%)**	
** Abiraterone**	9 (5%)
** Enzalutamide**	9 (5%)
** Pembrolizumab**	3 (2%)
** Darolutamide**	2 (1%)
** Apalutamide**	1 (1%)

Abbreviations: ARPI, androgen receptor pathway inhibitor; mCRPC, metastatic castration-resistant prostate cancer; PSA, prostate-specific antigen.

### Treatment exposure

The median number of prior mCRPC treatments was 3. Nearly all patients had received at least one prior ARPI (*n* = 180, 98%) with 57% receiving 2 or more ARPI. Additionally, the majority of patients received chemotherapy (85%), with 47% receiving 2 or more prior lines of taxane chemotherapy. Thirty-seven (20%) patients received prior radium-223. Overall, 21 patients (11%) received ^177^Lu-PSMA-617 with concurrent ARPI. With regards to definitive local therapy, 85 (46%) patients had prior prostatectomy and 97 (53%) had prostate radiation.

### Genetic testing and genomic associations

Overall, 50% of testing was performed on tissue with the remainder of testing performed on blood (Table S1). The majority of genetic testing was performed on tumor samples collected during CRPC (65%) and within 2 years of initiation of ^177^Lu-PSMA-617 (64%). The most commonly altered genes in the total cohort including *TP53* (51%), *AR* (40%), *TMPRSS2* (18%), and *PTEN* (16%) (Table S2). Overall, 109 patients had at least one genomic alteration in a TSG and 61 patients had at least one alteration in a HRR gene.

**Figure 1.**
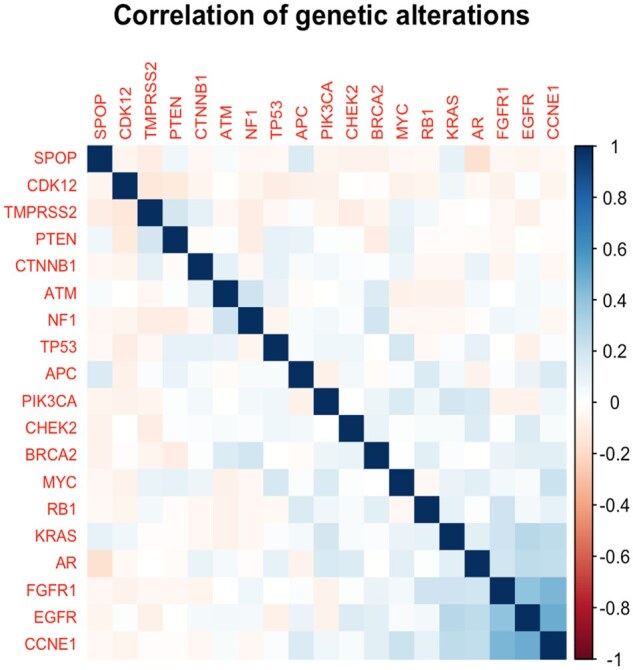
Correlation among 20 most frequently observed genetic alterations. Shading is based on the Matthew’s correlation coefficient among observed alterations. Gene pairs with darker shades of blue are more frequently observed to co-occur.

Correlation among the 20 most frequently observed genetic alterations is displayed (Figure 1). We observed associations between *EGFR*, *FGFR1*, and *AR* alterations. Among the 13 patients with an *EGFR* alteration, 11 also had an *AR* alteration (*R* = 0.25). Among the 10 patients with an *FGFR1* alteration, 8 also had an *AR* alteration (*R* = 0.20). Among the 10 patients with an *FGFR1* alteration, 5 also had an *EGFR* alteration (*R* = 0.40).

### PSA, PFS, and OS outcomes in the overall population

**Figure 2.**
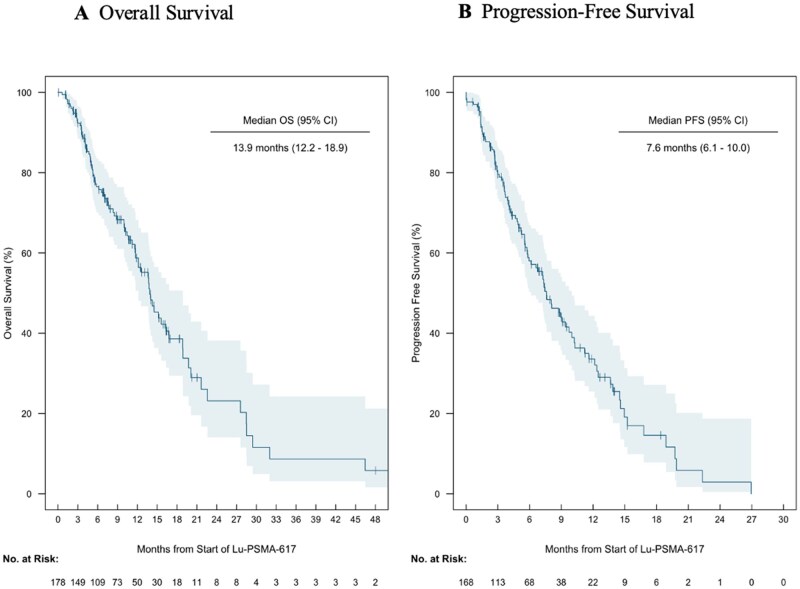
Kaplan–Meier plot of overall survival (A) and progression-free survival (B) for patients receiving ^177^Lu-PSMA-617 therapy. Shading represents the 95% confidence interval for the survival probability at each time point.

In the overall population, the PSA_50_ was 49% and PSA_90_ was 16%. The median follow-up for the entire cohort was 7.4 months. The median OS and PFS with ^177^Lu-PSMA-617 were 13.9 (95% CI 12.2-18.9) and 7.6 (95% CI 6.1-10.0) months, respectively (Figure 2). In patients with PSA_50_, the median OS and PFS were 18.9 (95% CI 16.3-32.0) and 12.5 months (95% CI 9.4-16.8), respectively. In patients without a PSA_50_, the median OS and PFS were 8.5 (95% CI 7.0-12.6) and 5.2 months (95% CI 3.4-6.6), respectively. It is notable that 7 patients experienced an undetectable PSA level (defined as PSA <0.20 ng/mL) after receiving ^177^Lu-PSMA-617. The median treatment time to achieve PSA nadir was 71 days and the mean time to achieve PSA nadir was 96 days. Since 177Lu-PSMA-617 is administered every 45 days, the PSA nadir was observed after roughly the completion of 2 treatment cycles.

### PSA, PFS, and OS outcomes in the overall population in molecular subgroups

Genetic alterations associated with PSA response (Figure S2), PSA_90_ (Table S3), OS (Figure S3), PFS (Figure S4), and PSA_50_ (Table 2, Figure S5) were evaluated. While in the overall population, the presence of alterations in *AR* was not associated with a lower PSA_50_ response rate (41% vs 55%, OR 0.57, 95% CI 0.30-1.06, *P* = .09), among patients with CRPC sequenced tumors, AR alterations were associated with lower PSA_50_ to ^177^Lu-PSMA-617 (38% vs 60%, OR 0.41, 95% CI 0.19-0.89, *P* = .03). AR alterations were persistently lower when we limited the analysis to patients that had genetic testing performed within 2 years of starting ^177^Lu-PSMA-617 therapy (37% vs 59%; *P* = .04; OR 0.40 [0.18-0.92]). Several other alterations showed trends toward lower PSA_50_ response rates, though these did not reach statistical significance: *CDK12* (25% vs 51%, OR 0.33, 95% CI 0.09-1.25, *P* = .13), *FGFR1* (20% vs 51%, OR 0.24, 95% CI 0.05-1.18, *P* = .10), *KRAS* (14% vs 50%, OR 0.17, 95% CI 0.02-1.40, *P* = .12), and *EGFR* (25% vs 51%, OR 0.33, 95% CI 0.09-1.25, *P* = .13). Conversely, all patients with *FOXA1* alterations (*n* = 5) achieved PSA_50_ response compared to 47% without this alteration, suggesting a potential benefit (*P* = .03). Other genes of interest including *SPOP* mutations (present in 4% of patients, 71% vs 48%, *P* = .27) and *ATM* alterations (present in 9% of patients, 56% vs 48%, *P* = .60) showed no significant association with PSA_50_ response rates. For PSA_90_ responses, the presence of *TP53* (10% vs 24%, OR 0.35, CI 0.14-0.85, *P* = .02) was associated with a lower PSA_90_. The presence of *NF1* was associated with an increased PSA_90_ response rate (50% vs 15%, OR 5.78, CI 1.35-24.77, *P* = .03).

**Table 2.** Percentages of PSA_50_ response in altered and non-altered groups. Bolded values are statistically significant.

Gene	Altered PSA_50_ response		Non-altered PSA_50_ response		*P*-value	OR (95% CI)
	Number of responder/Number Evaluable	%	Number of responder/Number Evaluable	%		
**Any TSG**	43/97	44.3	37/67	55.2	.20	0.65 (0.35-1.21)
**HRR**	28/58	48.3	52/106	49.1	1.0	0.97 (0.51-1.84)
***TP53***	38/83	45.8	42/81	51.9	.53	0.78 (0.43-1.44)
***AR***	29/71	40.9	51/93	54.8	.09	0.57 (0.30-1.06)
***TMPRSS2***	17/30	56.7	64/135	47.4	.54	1.37 (0.61-3.06)
***PTEN***	12/26	46.2	68/138	49.3	.83	0.88 (0.38-2.04)
***BRCA2***	10/17	58.8	70/147	47.6	.45	1.57 (0.57-4.35)
***ATM***	9/16	56.3	71/148	48.0	.60	1.39 (0.49-3.94)
***CHEK2***	6/15	40.0	74/149	49.7	.59	0.68 (0.23-2.00)
***CDK12***	3/12	25.0	77/152	50.7	.13	0.33 (0.09-1.25)
***EGFR***	3/12	25.0	77/152	50.7	.13	0.33 (0.09-1.25)
***APC***	6/11	54.6	74/153	48.4	.76	1.28 (0.38-4.38)
***PIK3CA***	4/10	40.0	76/154	49.4	.75	0.68 (0.19-2.52)
***FGFR1***	2/10	20.0	78/154	50.6	.10	0.24 (0.05-1.18)
***CTNNB1***	5/8	62.5	75/156	48.1	.49	1.80 (0.42-7.79)
***NF1***	**7/8**	**87.5**	**73/156**	**46.8**	**.03**	**7.96 (0.96**-**66.23)**
***SPOP***	5/7	71.4	75/157	47.8	.27	2.73 (0.52-14.51)
***RB1***	2/7	28.6	78/157	49.7	.44	0.41 (0.08-2.15)
***KRAS***	1/7	14.3	79/157	50.3	.12	0.17 (0.02-1.40)
***MYC***	2/7	28.6	78/157	49.7	.44	0.41 (0.08-2.15)
***BRCA1***	2/6	33.3	78/158	49.4	.68	0.51 (0.09-2.88)
***CCNE1***	1/6	16.7	79/158	50.0	.21	0.20 (0.02-1.75)
***ARID1A***	4/5	80.0	76/159	47.8	.20	4.37 (0.48-39.95)
***FOXA1***	**5/5**	**100.0**	**75/159**	**47.2**	**.03**	Inf (0.0-Inf)
***KMT2D***	2/5	40.0	78/159	49.1	1.0	0.69 (0.11-4.23)

Abbreviations: CI, confidence interval; NA, not applicable; OR, odds radio; PSA, prostate-specific antigen; TSG, tumor suppressor gene.

**Figure 3.**
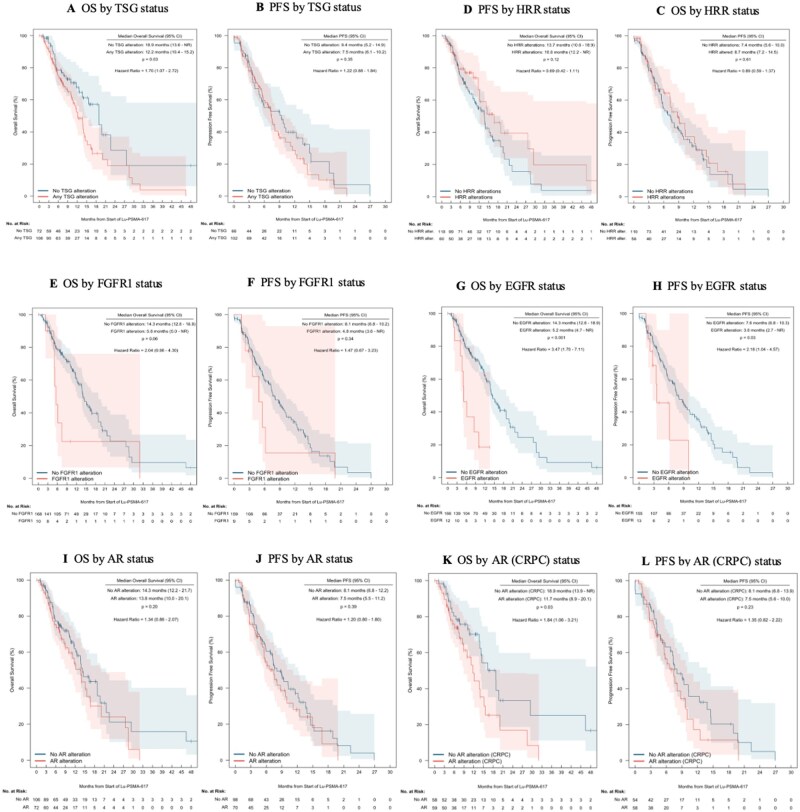
Kaplan–Meier plot of overall survival and progression-free survival by mutation status. (A) Overall survival by tumor suppresser gene alteration status. (B) Progression-free survival by tumor suppresser gene alteration status. (C) Overall survival by homologous recombination repair alteration status. (D) Progression-free survival by homologous recombination repair alteration status. (E) Overall survival by *EGFR/FGFR1* alteration status. (F) Progression-free survival by *EGFR/FGFR1* alteration status. (G) Overall survival by *AR* alteration status. (H) Progression-free survival by *AR* alteration status. OS = overall survival; PFS = progression-free survival; TSG = tumor suppressor gene; HRR = homologous recombination repair; AR = androgen receptor; Shading represents the 95% confidence interval for the survival probability at each time point.

TSG alterations were present in 109 (60%) patients. While patients with TSG alterations showed no significant difference in PSA_50_ response rates (44% vs 55%, OR 0.65, 95% CI 0.35-1.21, *P* = .20), they demonstrated significantly lower PSA_90_ response rates (10% vs 25%, OR 0.34, 95% CI 0.14-0.80, *P* = .02). Similarly, TSG alterations were not associated with differences in PFS (median 7.5 vs 9.4 months, HR 1.22, 95% CI 0.88-1.84, *P* = .35, Figure 3A) but were associated with significantly shorter median OS (12.2 vs 18.9 months, HR 1.70, 95% CI 1.07-2.72, *P* = .03, Figure 3B). The most common mutation present in non-responders to therapy was TP53 (54%, Table S4).

Homologous recombination repair (HRR) gene alterations were present in 61 (33%) patients (Figure 3C and D). PSA_50_ rates were similar between patients with and without HRR alterations (48% vs 49%, respectively). Among patients with HRR alterations, 30% (*n* = 18/61) had received prior PARP inhibitor therapy, with comparable PSA_50_ response rates between those with prior PARP inhibitor exposure (56%, *n* = 10/18) and those without (45%, *n* = 18/40). No significant differences were observed in survival outcomes between patients with and without HRR alterations, with median OS of 16.8 vs 13.7 months (HR 0.69, 95% CI 0.42-1.11, *P* = .12) and median PFS of 8.7 vs 7.4 months (HR 0.89, 95% CI 0.59-1.37, *P* = .61).

*FGFR1* mutations were observed in 10 patients (Figure 3E and F). Patients with *FGFR1* alterations showed a trend toward lower PSA_50_ rates that did not reach statistical significance (20% vs 51%, OR 0.24, 95% CI 0.05-1.18, *P* = .10). There was no significant difference in PFS (median 4.8 vs 8.1 months, HR 1.47, 95% CI 0.67-3.23, *P* = .34), and OS exhibited a strong non-significant trend toward shorter OS in *FGFR1*-altered patients (median 5.6 vs 14.3 months, HR 2.04, 95% CI 0.96-4.40, *P* = .06).

*EGFR* mutations were observed in 13 patients (Figure 3G and H), which consisted of 10 amplifications, 1 splice variant, 1 p. E114K, and 1 unknown. Patients with *EGFR* mutations showed a non-significant trend toward lower PSA_50_ rates (25% vs 51%, OR 0.33, 95% CI 0.09-1.25, *P* = .13) but demonstrated significantly shorter PFS (3.6 vs 7.6 months, HR 2.18, 95% CI 1.04-4.57, *P* = .03) and significantly shorter OS (5.2 vs 14.3 months, HR 3.47, 95% CI 1.70-7.11, *P* < .001).

In the total cohort, *AR* alterations were present in 73 (40%) patients (Figure 3I and J), including *AR* mutations (*n* = 31) and *AR* amplifications (*n* = 36). While the presence of *AR* alterations was associated with a lower PSA_50_ response rate among CRPC tumors (38% vs 60%, OR 0.41, 95% CI 0.19-0.89, *P* = .03), no significant differences were observed in survival outcomes between patients with and without *AR* alterations, with median PFS of 7.5 vs 8.1 months (HR 1.20, 95% CI 0.80-1.80) and median OS of 13.8 vs 14.3 months (HR 1.34, 95% CI 0.86-2.07). No significant differences were observed in PSA_50_ (OR 2.23, 95% CI 0.82-6.10, *P* = .14), PFS (HR 0.69, 95% CI 0.35-1.36) or OS (HR 0.99, 95% CI 0.47-2.07) between AR alteration subtypes.

For MSI-high patients (*n* = 10), 70% had a PSA_50_ response, median PFS was 5.5 months, and median OS was 8.9 months. Of the MSI-high, 5 of 10 received an immune checkpoint inhibitor (ICI) prior to ^177^Lu-PSMA-617 whereas one patient received ICI after ^177^Lu-PSMA-617.

**Figure 4.**
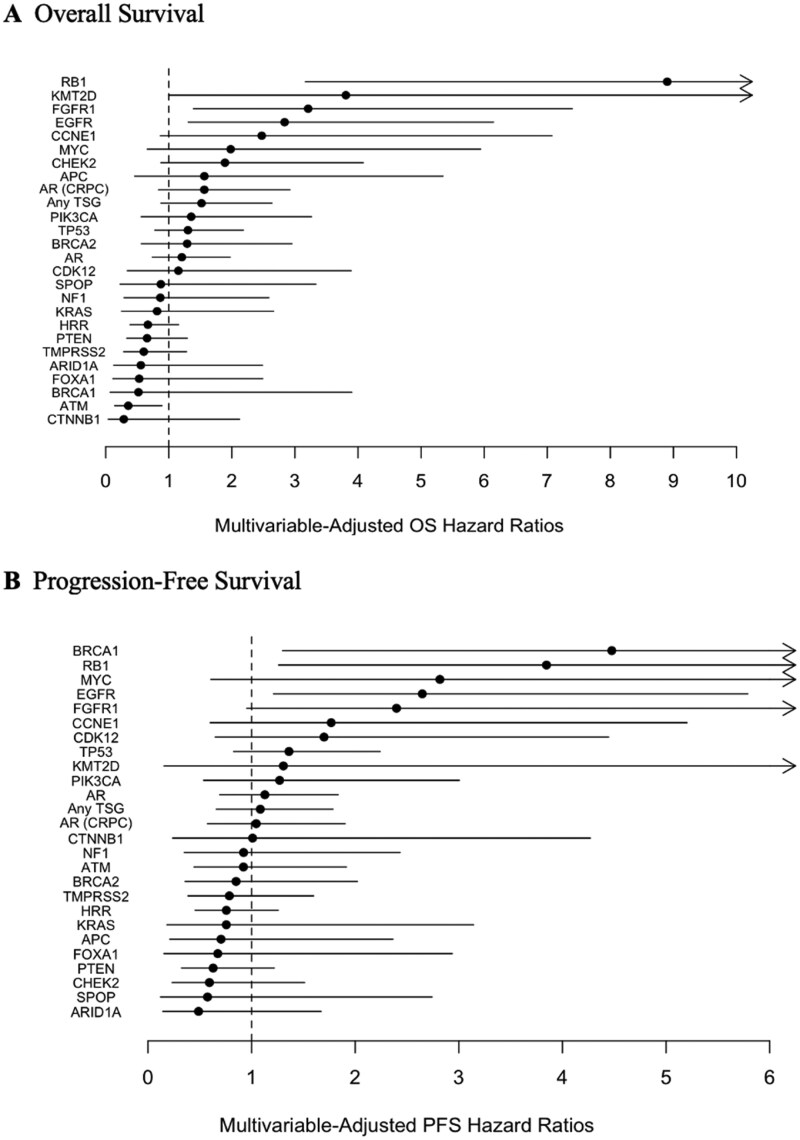
Multivariate-adjusted hazard ratios for overall survival (A) and progression-free survival (B). Points are estimated hazard ratios between altered and non-altered groups adjusted for age, prostate specific antigen at treatment start, visceral metastases. Lines are 95% confidence intervals for these hazard ratios. All genes with more than 5 altered patients are shown.

Multivariable analyses for OS and PFS were performed, adjusting for key clinical variables (Figure 4; Figures S2-S4). Mutations that were significantly associated with worse OS in this multivariable analysis included *RB1*, *FGFR1*, *EGFR*, and *KMT2D* while *ATM* was associated with improved OS in this multivariable analysis. Mutations that were associated with significantly worse PFS included *BRCA1*, *RB1*, and *EGFR*.

## Discussion

This study represents a detailed analyses of molecular predictors of response to ^177^Lu-PSMA-617 in patients with PSMA PET positive mCRPC. Leveraging the PROMISE database, we analyzed data from 183 patients across 11 academic institutions. This robust database allowed for an examination of the interplay between molecular alterations and treatment outcomes. This approach, combining clinical characteristics, treatment histories, and genomic profiles, provides a unique opportunity to identify potential markers for ^177^Lu-PSMA-617 response and resistance in a real-world setting. Our findings contribute to the growing body of knowledge about predictors of response to therapy and highlight the power of collaborative, data-driven approaches in advancing precision medicine for mCRPC.^22^

In our study, we reported a median PFS of 7.6 and OS of 13.9 months in patients who received ^177^Lu-PSMA-617. This is comparable to outcomes reported in the experimental arm of the VISION trial (PFS = 8.7 months, OS of 15.3 months).^2^ The prior treatments in our dataset mirror those of the VISION trial, which reported that 213 patients (39%) received >1 ARPI and 220 patients (40%) received >1 taxane in the ^177^Lu-PSMA-617 (*n* = 551) arm. It is important to note that our population was heavily pretreated and reflects the clinical landscape when ^177^Lu-PSMA-617 first entered the treatment landscape in the United States as a standard of care option, representing outcomes in patients who received LuPSMA as a later line therapy after exhausting multiple standard treatment options, which may impact the observed efficacy profile compared to potential earlier use in the disease course.

Among patients with TSG alterations, we observed worse PSA_90_ responses and OS. TSG alterations are strongly associated with aggressive disease and the development of treatment-resistant phenotypes, often leading to mCRPC. This is consistent with data associating TSG mutations with shorter PSA_50_, PFS, and OS in patients receiving ^177^Lu-PSMA-617.^16,17^ This interplay underscores the complexity of prostate cancer progression and the need for alternative targeting strategies in advanced disease. Notably, while VISION trial imaging criteria aimed to exclude patients with PSMA-low/negative metastases (often associated with these phenotypes), our findings suggest these TSG alterations may influence outcomes even before driving overtly PSMA-negative disease or may do so in a sub-clonal manner below PET detection threshold.

The relationship between *AR* alterations and PSMA expression in prostate cancer is complex. We demonstrated that among CRPC sequenced tumors, patients with *AR* alterations were less likely to exhibit a PSA_50_ response; however, there was not a significant difference in PFS. A weak, though not significant association was observed with OS. We acknowledge tumor genomic features can evolve throughout multiple lines of cancer treatment, so we subsequently limited our analysis to patients who received genetic testing within 2 years of starting ^177^Lu-PSMA-617, PSA_50_ responses were persistently lower in the AR alterations group. Other molecular studies have demonstrated associations between high PSMA expression and the presence of *AR* alterations, but this has not always correlated with treatment response.^23^^,^^24^ Furthermore, ctDNA molecular analyses from the PSMAFore trial of taxane-naïve patients who received ^177^Lu-PSMA-617 versus ARPI demonstrated that the presence of AR amplifications was associated with shorter PFS.^25^ These findings should be interpreted with caution, as they require correction for ctDNA fraction. AR amplifications have been shown to be prognostic of worse outcomes across multiple treatment settings.^26–28^ These observations highlight the intricate relationship between AR signaling, PSMA expression, and treatment response, suggesting that the efficacy of PSMA-targeted therapies may be influenced by the underlying AR biology and prior exposure to AR inhibitors.

We observed similar outcomes among patients with and without *HRR* alterations, which adds to the growing body of literature and extends other preliminary observations.^29^^,^^30^ We reported a trend toward reduced PSA_50_ responses in patients with *CDK12* alterations (25% vs 51%, *P* = .13), which is consistent with prior reported data by Sartor et al.^2^  *CDK12* plays a critical role in transcriptional regulation and DNA damage response, but is associated with more aggressive disease and worse outcomes.^16^^,^^31^^,^^32^ Additionally, while we did not observe a statistically significant difference in PSA_50_ response rates between patients with and without *ATM* alterations, responses were numerically higher in *ATM*-altered patients, a finding that may be limited by our sample size. *ATM* is a pivotal DNA damage repair mechanism. Raychaudhuri et al. reported excellent clinical outcomes in patients with *ATM* alterations treated with ^177^Lu-PSMA-617.^16^ This was thought to be attributed to the radiosensitivity that has been shown *ATM* mutations.

Our analysis revealed that *EGFR* alterations were associated with worse outcomes, trending toward lower PSA_50_ with significantly shorter OS. *EGFR* has been associated with poor prognosis in prostate cancer.^33^ Nastaly et al. demonstrated that *EGFR* overexpression was associated with shorter metastasis-free survival and was an independent factor of OS.^34^ These findings collectively underscore the importance of *EGFR* alterations as potential biomarkers for resistance to ^177^Lu-PSMA-617 and highlight the need for further investigation.

Our work demonstrates a trend toward favorable outcomes with the use of ^177^Lu-PSMA-617 in patients with *FOXA1* alterations. This was a statistically significant outcome (*P* = .03), although this finding was based off only 5 patients containing *FOXA1*. *FOXA1* is essential for *AR* signaling, facilitating *AR* binding to DNA and enhancing the expression of *AR* target genes. In prostate cancer, *FOXA1* contributes to *AR* signaling even in low androgen environments.^35–38^ This suggests that tumors with *FOXA1* alterations may maintain higher levels of *AR*-regulated genes, including PSMA.^39^ Consequently, these tumors could potentially respond better to ^177^Lu-PSMA-617. The persistent *AR* signaling facilitated by altered *FOXA1* might ensure higher target availability for PSMA-directed therapies, potentially leading to improved outcomes.

Our findings highlight *NF1* alterations as a potential biomarker associated with improved response to ^177^Lu-PSMA-617. *NF1* is a TSG that negatively regulates the *RAS* pathway, and its loss leads to constitutive activation of downstream signaling cascades, including the *MAPK* and *PI3K/AKT* pathways. The enhanced response of *NF1*-altered tumors to radionuclide therapy may result from increased PSMA expression, greater radiosensitivity due to DNA damage response defects, or alterations in the tumor microenvironment. This unexpected association merits further investigation and could inform combination strategies with *RAS* pathway inhibitors in select patients.

As demonstrated in Figure 1, we reported a co-occurrence of *AR* alterations with *EGFR* and *FGFR1* alterations. This suggests complementary resistance mechanisms in prostate cancer. These growth factor pathway alterations may work alongside *AR* signaling changes to promote tumor survival despite ^177^Lu-PSMA-617 therapy. This pattern could explain why patients with these combined genomic features showed poorer treatment outcomes and points to potential combination treatment strategies targeting multiple pathways simultaneously.

While our study provides valuable insights into genetic biomarkers of ^177^Lu-PSMA-617 response, several limitations should be acknowledged. As a retrospective analysis conducted at academic medical centers, our findings may be subject to selection bias and limited generalizability to community practice settings. Since genomic data were extracted from sequencing reports, the specific alterations present were limited to those represented on sequencing reports. Specific genomic sequencing panels that were utilized varied by institution and were not standardized. Additionally, the relatively small sample size, especially for rare genetic subtypes of mCRPC, and the limited ability to adjust for a large range of clinical variables limited the ability to perform a granular multivariable analysis. Due to small sample sizes, we had limited power to detect alterations associated with PSA_50_ response at a statistical significance level of *P* < .05, but we will highlight alterations that exhibited noteworthy differences in PSA_50_ response across alteration status but were only significant at the *P* < .15 level. These include alterations in the following genes: *CDK12, EGFR, FGFR1, NF1, KRAS, FOXA1*, and *TSG (TP53, RB1*, or *PTEN*).

## Conclusions

This multi-institutional study provides valuable insights into the molecular landscape of mCRPC treated with ^177^Lu-PSMA-617. Our findings suggest that alterations in specific genes, notably *AR*, TSG alterations, *EGFR*, *FGFR1*, and others, may influence the efficacy of ^177^Lu-PSMA-617. The presence of these alterations was associated with lower PSA response rates and shorter survival, highlighting their potential as biomarkers for patient selection and prognostication. Our results underscore the complex interplay between genomic alterations and treatment outcomes in mCRPC. As we continue to refine our understanding of the molecular determinants of ^177^Lu-PSMA-617 response, we move closer to optimizing the use of ^177^Lu-PSMA-617 in clinical practice, potentially enhancing treatment efficacy and patient outcomes through more precise, genomically guided application of this therapy.

## Supplementary Material

oyaf358_Supplementary_Data

## Data Availability

The data underlying this article are available in the article and in its online supplementary material.
