# Supplementary material for: Genomic alterations and associated outcomes in patients with PSMA-positive metastatic castration-resistant prostate cancer treated with 177Lu-PSMA-617
Source: Oncologist. 2025 Oct 27;30(11):oyaf358. doi: 10.1093/oncolo/oyaf358 (PMC12622373; doi:10.1093/oncolo/oyaf358)

**Supplemental Figures.**

**Supplement Figure S1.** Consort diagram. NGS=Next generation sequencing; mCRPC=Metastatic castration-resistant prostate cancer.


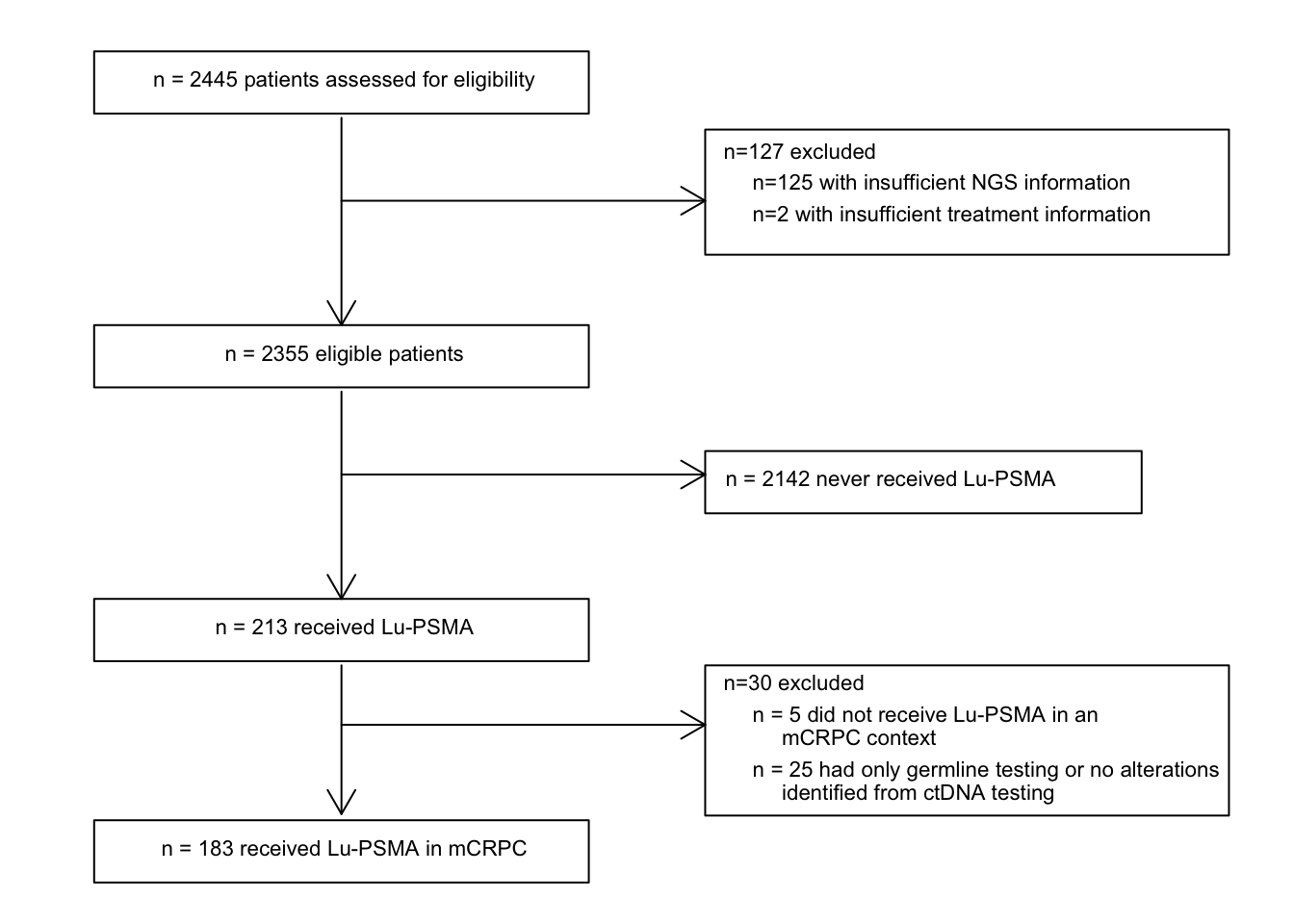


**Supplement Figure S2.** Univariate analyses for PSA response. This figure plots difference in PSA percentage decline between altered group and wild type group. Points represent the observed difference and the lines indicate a 95% confidence interval for this difference. PSA=Prostate specific antigen.


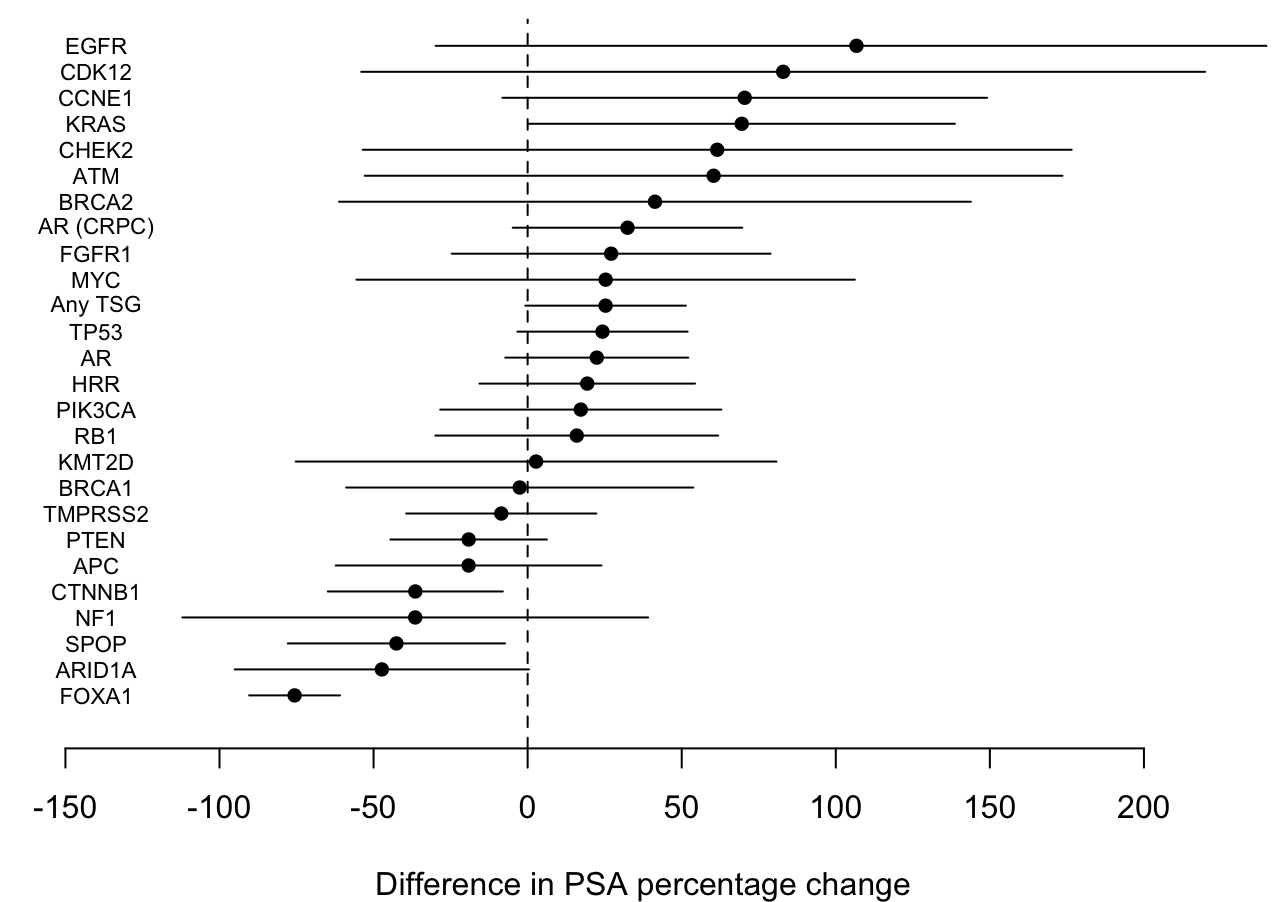


**Supplement Figure S3.** Univariate analysis for overall survival. Points are estimated hazard ratios between altered groups and wild type groups. Lines are 95% confidence intervals for these hazard ratios. All genes with more than 5 altered patients are shown.


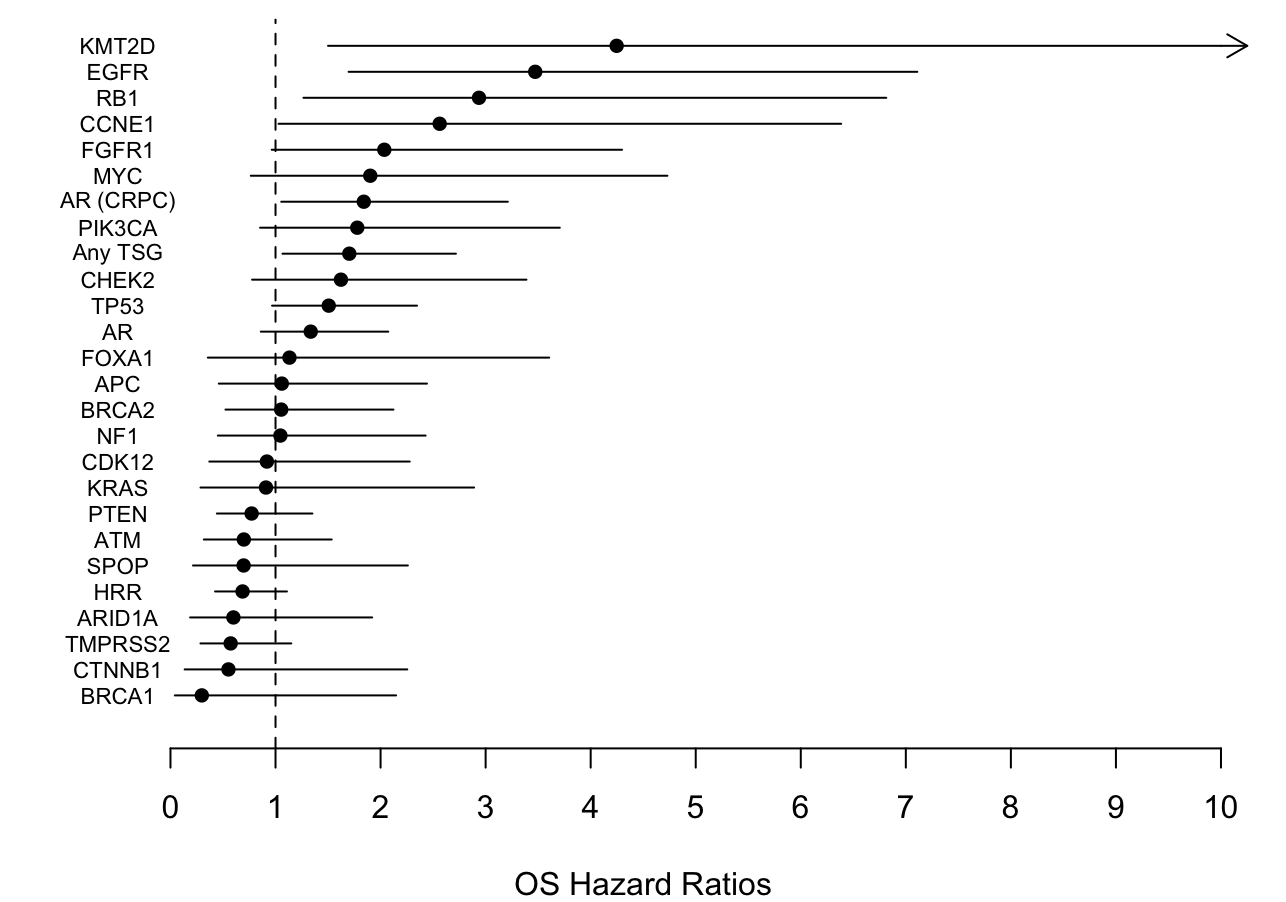


**Supplement Figure S4.** Univariate analysis for progression-free survival. Points are estimated hazard ratios between altered groups and wild type groups. Lines are 95% confidence intervals for these hazard ratios.


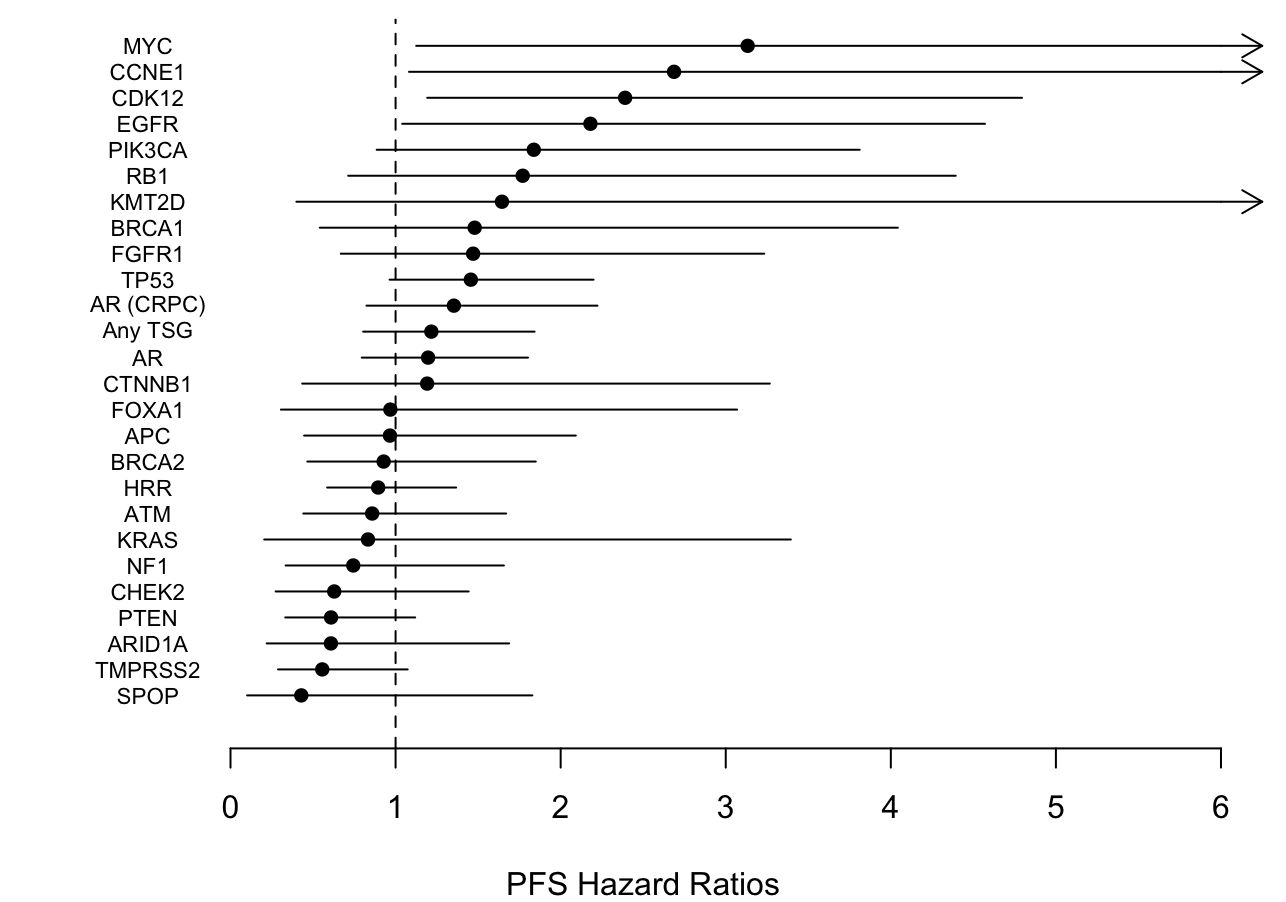


**Supplemental Figure S5.** PSA_50_ response odds ratios by genetic alteration. Points are estimated odds ratios between altered groups and wild type groups. Lines are 95% confidence intervals for these odds ratios.


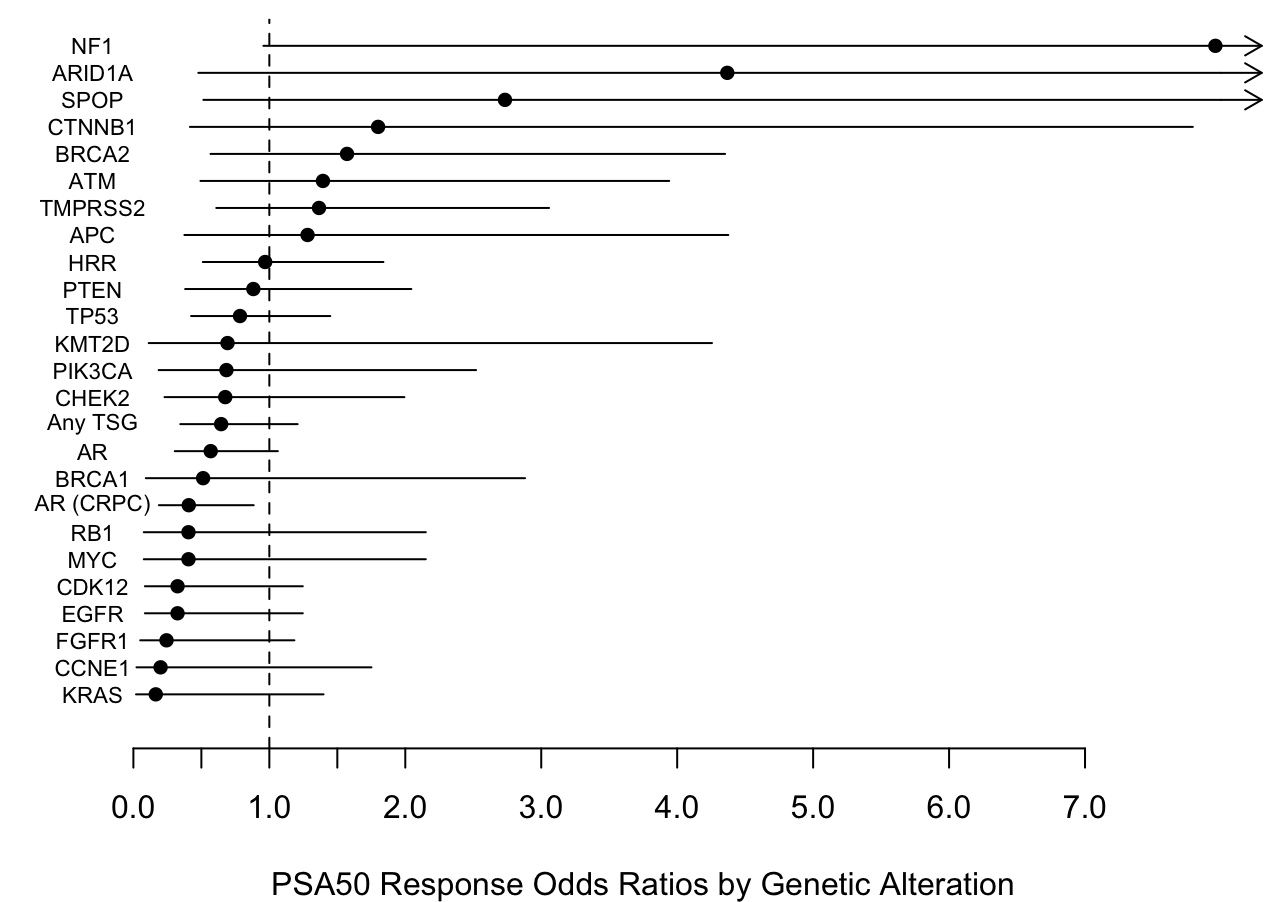

Supplement: oyaf358_Supplementary_Data [file oyaf358_supplementary_data.zip › Pluvicto Supplemental Figures 09Jun2025 CLEAN.docx]
