# Supplementary material for: Genomic alterations and associated outcomes in patients with PSMA-positive metastatic castration-resistant prostate cancer treated with 177Lu-PSMA-617
Source: Oncologist. 2025 Oct 27;30(11):oyaf358. doi: 10.1093/oncolo/oyaf358 (PMC12622373; doi:10.1093/oncolo/oyaf358)
Supplement: oyaf358_Supplementary_Data [file oyaf358_supplementary_data.zip › Pluvicto Supplemental Tables 09Jun2025 CLEAN.docx]

**Supplemental Tables.**

**Supplement Table S1.** Specimen source for sequencing, disease context at time of sequencing, and timing of sequencing from ^177^Lu-PSMA-617 treatment initiation.

| Specimen Source, Number (%), N=183 | |
| --- | --- |
| Bone | 12 (7%) |
| ctDNA | 89 (49%) |
| Liver | 6 (3%) |
| Lung | 5 (3%) |
| Lymph Nodes | 21 (12%) |
| Other | 6 (3%) |
| Prostate | 41 (22%) |
| Unknown | 3 (2%) |
| Disease context in which specimen was collected, Number (%) | |
| CRPC | 119 (65%) |
| HSPC | 57 (31%) |
| Unknown | 7 (4%) |
| Timing of last specimen before ^177^Lu-PSMA-617 start, Number (%) | |
| <100 days | 38 (21%) |
| 100 days – 1 year | 37 (20%) |
| 1-2 years | 43 (24%) |
| 2-3 years | 14 (8%) |
| 3-5 years | 15 (8%) |
| $\geq$5 years | 17 (9%) |
| After ^177^Lu-PSMA-617 start | 15 (8%) |
| Missing | 4 (2%) |

CRPC=Castration-resistant prostate cancer; HSPC=Hormone sensitive prostate cancer; ctDNA=Circulating tumor DNA

**Supplement Table S2**. Distrubution of genomic alterations in the total cohort.

| **Gene Alteration** | **Frequency (N=183)** |
| --- | --- |
| TP53 | 94 (51%) |
| AR | 73 (40%) |
| TMPRSS2 | 32 (18%) |
| PTEN | 30 (16%) |
| BRCA2 | 18 (10%) |
| ATM | 17 (9%) |
| CHEK2 | 16 (9%) |
| APC | 14 (8%) |
| EGFR | 13 (7%) |
| CDK12 | 12 (7%) |
| PIK3CA | 11 (6%) |
| FGFR1 | 10 (6%) |
| MYC | 9 (5%) |
| NF1 | 8 (4%) |
| CTNNB1 | 8 (4%) |
| SPOP | 7 (4%) |
| RB1 | 7 (4%) |
| KRAS | 7 (4%) |
| CCNE1 | 7 (4%) |
| KMT2D | 6 (3%) |
| FOXA1 | 6 (3%) |
| BRCA1 | 6 (3%) |
| ARID1A | 6 (3%) |
| Any TSG | 109 (60%) |
| Any HRR | 61 (33%) |

**Supplement Table S3.** Percentages of PSA90 response in altered versus non-alterated groups. Bolded values are statistically significant.

| **Genes** | **Altered PSA_90_ Response** | | **Non-Altered PSA_90_ Response** | | **p-value** | **OR (95% CI)** |
| --- | --- | --- | --- | --- | --- | --- |
|  | **Number of responder/Number Evaluable** | **%** | **Number of responder/Number Evaluable** | **%** |  |  |
| **Any TSG** | **10/97** | **10.3** | **17/67** | **25.4** | **0.02** | **0.34 (0.14 – 0.80)** |
| HRR | 11/58 | 19.0 | 16/106 | 15.1 | 0.52 | 1.32 (0.57 – 3.06) |
| **TP53** | **8/83** | **9.6** | **19/81** | **23.5** | **0.02** | **0.35 (0.14-0.85)** |
| AR | 10/71 | 14.1 | 17/93 | 18.3 | 0.53 | 0.73 (0.31-1.72) |
| **AR(CRPC)** | **5/58** | **8.6** | **13/50** | **26.0** | **0.02** | **0.27 (0.09 – 0.82)** |
| TMPRSS2 | 5/29 | 16.7 | 22/135 | 16.3 | 1.0 | 1.07 (0.37-3.11) |
| PTEN | 4/26 | 15.4 | 23/138 | 16.7 | 1.0 | 0.91 (0.29-2.89) |
| BRCA2 | 4/17 | 23.5 | 23/147 | 15.6 | 0.49 | 1.66 (0.50-5.54) |
| ATM | 5/16 | 31.3 | 22/148 | 14.9 | 0.15 | 2.60 (0.82-8.22) |
| CHEK2 | 2/15 | 13.3 | 25/149 | 16.8 | 1.0 | 0.76 (0.16-3.59) |
| CDK12 | 0/12 | 0.0 | 27/152 | 17.8 | 0.22 | 0.0 (0.0 - Inf) |
| EGFR | 0/12 | 0.0 | 27/152 | 17.8 | 0.22 | 0.0 (0.0 - Inf) |
| APC | 2/11 | 18.2 | 25/153 | 16.3 | 1.0 | 1.14 (0.23-5.58) |
| PIK3CA | 0/10 | 0.0 | 27/154 | 17.5 | 0.37 | 0.0 (0.0 – Inf) |
| FGFR1 | 1/10 | 10.0 | 26/154 | 16.9 | 1.0 | 0.55 (0.07-4.51) |
| CTNNB1 | 1/8 | 12.5 | 26/156 | 16.7 | 1.0 | 0.71 (0.08-6.05) |
| **NF1** | **4/8** | **50.0** | **23/156** | **14.7** | **0.03** | **5.78 (1.35-24.77)** |
| SPOP | 2/7 | 28.6 | 25/157 | 15.9 | 0.32 | 2.11(0.39-11.50) |
| RB1 | 0/7 | 0.0 | 27/157 | 17.2 | 0.60 | 0.0 (0.0 – Inf) |
| KRAS | 1/7 | 14.3 | 26/157 | 16.6 | 1.0 | 0.84 (0.10-7.27) |
| MYC | 1/7 | 14.3 | 26/157 | 16.6 | 1.0 | 0.84 (0.10-7.27) |
| BRCA1 | 1/6 | 16.7 | 26/158 | 16.5 | 1.0 | 1.02 (0.11-9.05) |
| CCNE1 | 0/6 | 0.0 | 27/158 | 17.1 | 0.59 | 0.0 (0.0 – Inf) |
| ARID1A | 2/5 | 40.0 | 25/159 | 15.7 | 0.19 | 3.57 (0.57-22.49) |
| **FOXA1** | **4/5** | **80.0** | **23/159** | **14.5** | **0.003** | **23.65 (2.53-221.16)** |
| KMT2D | 1/5 | 20.0 | 26/159 | 16.4 | 1.0 | 1.28 (0.14-11.91) |

PSA=Prostate specific antigen; TSG=Tumor suppressor gene, CI=confidence interval, OR=odds radio, NA=not applicable

Supplement Table S4. The most common genomic alterations present in the responders and non-responders groups.

| Most common genomic alterations | Responders Group (n=80) | Non-responders group (n=84) |
| --- | --- | --- |
| 1 | TP53: 38 (48%) | TP53: 45 (54%) |
| 2 | AR: 29 (36%) | AR: 42 (50%) |
| 3 | TMPRSS2: 16 (20%) | PTEN:  14 (17%) |
| 4 | PTEN:  12 (15%) | TMPRSS2: 13 (16%) |
| 5 | BRCA2:  10 (13%) | CDK12: 9 (11%)  CHEK2: 9 (11%)  EGFR:   9 (11%) |
| 6 | ATM:  9 (11%) |  |
| 7 | NF1: 7 (9%) |  |
| 8 | APC: 6 (8%)  CHEK2: 6 (8%) | FGFR1:  8 (10%) |
| 9 |  | ATM:  7 (8%)  BRCA2: 7 (8%) |
| 10 | CTNNB1: 5 (6%) FOXA1:    5 (6%)  SPOP:      5 (6%) |  |
| 11 |  | KRAS: 6 (7%)  PIK3CA: 6 (7%) |
